# Genome‐wide association studies in Crohn's disease: Past, present and future

**DOI:** 10.1002/cti2.1001

**Published:** 2018-01-31

**Authors:** Bram Verstockt, Kenneth GC Smith, James C Lee

**Affiliations:** ^1^ Translational Research in Gastrointestinal Disorders (TARGID) ‐ IBD Department of Chronic Diseases, Metabolism and Ageing (CHROMETA) KU Leuven Leuven Belgium; ^2^ Department of Gastroenterology and Hepatology University Hospitals Leuven Leuven Belgium; ^3^ Department of Medicine University of Cambridge School of Clinical Medicine Addenbrooke's Hospital Cambridge UK

**Keywords:** Crohn's disease, GWAS, Pharmacogenetics, prognosis, susceptibility

## Abstract

Over the course of the past decade, genome‐wide association studies (GWAS) have revolutionised our understanding of complex disease genetics. One of the diseases that has benefitted most from this technology has been Crohn's disease (CD), with the identification of autophagy, the IL‐17/IL‐23 axis and innate lymphoid cells as key players in CD pathogenesis. Our increasing understanding of the genetic architecture of CD has also highlighted how a failure to suppress aberrant immune responses may contribute to disease development – a realisation that is now being incorporated into the design of new treatments. However, despite these successes, a significant proportion of disease heritability remains unexplained. Similarly, most of the causal variants at associated loci have not yet been identified, and even fewer have been functionally characterised. Because of the inarguable rise in the incidence of CD in regions of the world that previously had low disease rates, GWAS studies will soon have to shift from a largely Caucasian focus to include populations from other ethnic backgrounds. Future studies should also move beyond conventional studies of disease susceptibility into phenotypically driven ‘within‐cases’ analyses in order to explore the role of genetics in other important aspects of disease biology. These studies are likely to include assessments of prognosis and/or response to treatments and may be critical if personalised medicine is ever to become a reality.

## Introduction

**Figure 1:**
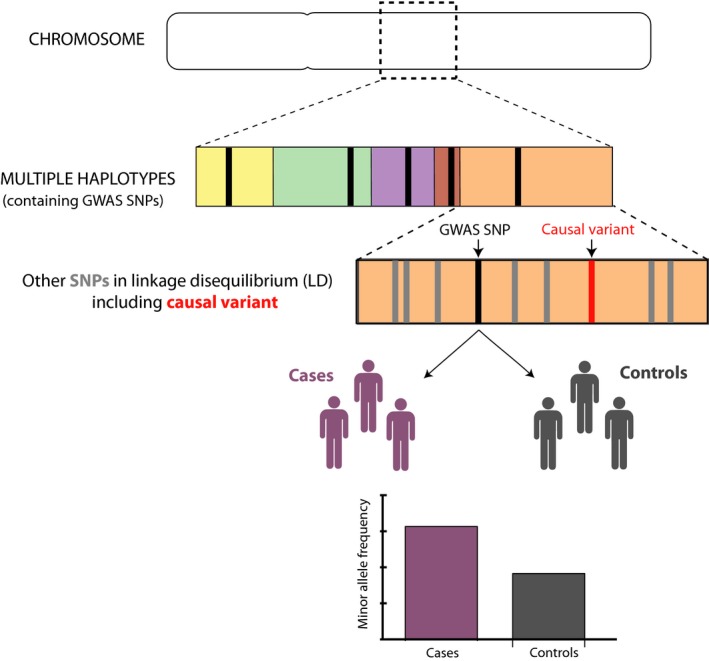
Basic principles of GWAS. GWAS has been made possible because of the haplotype structure of the human genome. Every chromosome consists of multiple haplotypes – regions that are inherited together during meiosis. Within each haplotype, there are typically many SNPs, which are co‐inherited within the larger genetic region, and thus, their alleles are inherited nonrandomly (i.e. they are in linkage disequilibrium). This means that it is possible to infer the genotypes at multiple SNPs within the haplotype (shown in grey) if the genotype at one or more SNPs is known. GWAS SNPs (shown in black) are selected so as to tag each haplotype, but where association is observed, they are unlikely to be the causal variant at the locus (shown in red). By genotyping SNPs from each haplotype in the genome in disease cases and healthy controls, it is possible to identify SNPs where the allele frequency is significantly different between the cases and controls, and which are associated with the disease.

Crohn's disease (CD) and ulcerative colitis (UC), collectively termed Inflammatory Bowel Disease (IBD), are some of the most extensively and successfully studied diseases in complex disease genetics. However, genetic studies in CD were not always successful. Initial efforts to identify the genetic determinants of CD were performed using linkage mapping and were largely disappointing, often producing weak or inconsistent signals.^1^ One exception to this was the discovery of *NOD2* as a major CD susceptibility gene^2^, ^3^, ^4^ – a finding that represents one of the few successes of linkage mapping across all diseases. The development of genome‐wide association studies (GWAS), which facilitate a hypothesis‐free comparison of allele frequencies at thousands of single nucleotide polymorphisms (SNPs) between cases and controls (Figure 1), transformed the study of CD genetics and led to the discovery of many CD susceptibility SNPs. Nonetheless, the overall number of hits identified in early, single cohort GWAS studies was typically modest (between 1 and 10) due to relatively small sample sizes and the resulting limitations in study power.^5^, ^6^, ^7^, ^8^ Fortunately, the combination of a high disease heritability and a strong collaborative spirit between research groups from around the world meant that subsequent meta‐analyses could include much larger numbers of samples and were accordingly far more successful in identifying the genetic determinants of IBD.^9^, ^10^, ^11^, ^12^ For example, in 2008, the first international meta‐analysis was performed by combining GWAS data from the UK, US and Franco‐Belgian IBD Genetics consortia^13^ – studies that had individually identified a total of 10 susceptibility loci.^5^, ^6^, ^8^ The gain in power that was afforded by combining these data sets led to the identification of 32 associated loci^13^ and highlighted the value of collaborating to create larger data sets for analysis. This study was unsurprisingly followed 2 years later by an even larger meta‐analysis – incorporating ~22 000 cases and ~29 000 controls from 13 different countries – in which 71 susceptibility loci were identified.^9^ Subsequent larger studies, which have taken advantage of more affordable genotyping approaches (such as the Illumina Immunochip), have increased this number even further, with 241 IBD susceptibility loci being confirmed in the most recent analysis.^14^ Such advances clearly demonstrate the power of GWAS to provide novel insights into disease pathogenesis, particularly when applied to an ever‐increasing sample size.^15^ Interestingly, the majority of these loci increase the risk of both CD and UC,^14^ and many of them alter susceptibility to other immune‐mediated diseases as well – including diseases that are known to be related to IBD, such as primary sclerosing cholangitis, psoriasis and ankylosing spondylitis, and others that were previously thought to be unrelated, such as type 1 diabetes and multiple sclerosis.^16^ Despite this undoubted success, however, it is important to note that most GWAS hits are only proxies for the true causal variant(s) at each locus (with which they are inherited through linkage disequilibrium). Indeed, it was the realisation that entire haplotypes could be interrogated by genotyping just one of their constituent SNPs that helped make GWAS possible in the first place, as it meant that genome‐wide genetic variation could be captured using a tractable number of variants (that could be incorporated into a single genotyping chip) (Figure 1). As such, to identify the functional consequences of these associated haplotypes – and the mechanism by which they alter disease risk – it will be important to identify the causal variant(s) that is ultimately responsible for the association. In some cases, this may be possible using statistical fine mapping – such as has recently been performed by the International Inflammatory Bowel Disease Genetic Consortium (IIBDGC)^17^ – but in others, some form of functional characterisation of the downstream effects of each SNP in a locus will be necessary.

Genetics alone will not provide all of the answers, but if combined with careful study of the downstream biology, it seems likely that new pathways involved in disease biology will be discovered – improving our understanding of disease pathogenesis and potentially providing new opportunities for therapeutic intervention.

## Unravelling disease pathogenesis – the genetic contribution

Although the pathophysiology of CD is not fully understood, the current hypothesis assumes a complex interplay between environmental, genetic and intestinal microbial factors, all of which interact with the host's immune system to result in a pathological auto‐inflammatory response directed towards the intestine.^18^ During the past decade, GWASs have identified multiple SNPs that – by virtue of their proximity to genes known to be involved in specific pathways – have led to the implication of these pathways in disease development. Some of these proposed pathophysiological mechanisms will now be discussed in more detail – especially those that have made important contributions to our current understanding of CD pathogenesis (Table 1).

**Table 1:** Summary of IBD GWAS hits involved in autophagy, the IL‐17/IL‐23 axis/type 3 innate lymphoid cells and the failure to suppress of aberrant immune responses

	Chromosome	SNP	*P* value	OR	AF	Candidate gene in/near locus
Autophagy	2	rs6752107	1.42E−73	1.25	0.55	*ATG16L1*
	5	rs11741861	2.94E−37	1.25	0.05	*IRGM*
	12	rs11564258	6.38E−29	1.33	0.025	*LRRK2*
	16	rs2066844	2.27E−217	2.00	0.06	*NOD2*
IL‐23/IL‐17 Axis Type 3 innate lymphoid cells	1	rs11581607	8.76E−175	0.46	0.05	*IL23R*,* IL12RB2*
	1	rs4845604	1.21E−17	0.88	0.13	*RORC*
	5	rs56167332	7.17E−50	1.17	0.35	*IL12B*
	6	rs1819333	6.76E−21	1.08	0.48	*CCR6*
	9	rs75900472	4.70E−48	1.16	0.13	*JAK2*
	12	rs11614178	2.22E−32	1.19	0.4	*IL22*,* IFNG*
	17	rs12942547	5.51E−22	1.1	0.55	*STAT3*,* STAT5A*,* STAT5B*
	19	rs11879191	5.27E−20	0.89	0.2	*TYK2*
	21	rs7282490	2.35E−26	1.1	0.39	*ICOSLG*
Failure to suppress aberrant immune responses	1	rs3024505	2.99E−50	1.22	0.19	*IL10*
	4	rs7657746	3.00E−13	1.1	0.20	*IL2*
	7	rs1077773	5.96E−9	0.93	0.48	*AHR*
	10	rs12722515	3.76E−10	1.1	0.85	*IL2RA*
	15	rs17293632	2.71E−20	1.11	0.14	*SMAD3*
	16	rs529866	1.73E−16	1.12	0.81	*SOCS1*
	17	rs12942547	5.51E−22	1.1	0.55	*STAT3*,* STAT5A*,* STAT5B*
	18	rs7240004	1.01E−10	0.94	0.34	*SMAD7*
	21	rs2284553	2.14E−16	1.12	0.60	*IL10RB*

Data are collated from Jostins *et al*.,^11^ Liu *et al*.,^12^ and Huang *et al*.^17^

Allele frequency (AF) refers to allele frequency in 1000 genomes CEU population of the allele for which odds ratio (OR) is reported.

### Autophagy

Prior to genetic studies that identified *ATG16L1* and *IRGM* as susceptibility loci for CD,^7^, ^19^ a role for autophagy in disease pathogenesis had not been envisaged. Autophagy is a well‐described and evolutionarily conserved process whereby cytoplasmic components are engulfed by double‐membraned vesicles (autophagosomes) that then fuse with lysozymes in order for their contents (protein aggregates and damaged organelles) to be degraded and recycled.^20^ Given the importance of intestinal microbes in CD pathogenesis, it is notable that autophagy also plays a role in clearance of intracellular micro‐organisms (a process known as ‘xenophagy’).^21^, ^22^ Moreover, autophagy has also been linked to endoplasmic reticulum (ER) stress^23^ – another process that has been implicated in intestinal inflammation and IBD.^24^ ER stress has been shown to induce autophagy, and autophagy reciprocally plays a role in resolving ER stress.^25^ Interestingly, Crohn's disease risk SNPs seem to be able to disrupt this balance, which can lead to intestinal inflammation.^26^

When autophagy genes were first associated with CD, the mechanism by which autophagy altered disease risk was unknown. Several groups therefore sought to investigate this by genetically targeting *ATG16L1* 27, 28, 29 – the first autophagy gene to be identified.^7^ Most of these studies used loss‐of‐function approaches (i.e. *ATG16L1* knockout or knock‐down) and while they provided interesting insights into the role of autophagy in intestinal inflammation, it was unclear as to how the results might relate to the risk variant identified by GWAS. This was because unlike most GWAS hits, which are typically noncoding and thus presumed to affect gene expression, the risk SNP in *ATG16L1* is a missense mutation that leads to a threonine to alanine substitution at position 300 (rs2241880, Thr300Ala or T300A).^7^ Missense mutations are generally assumed to affect protein structure or function due to the effect that an amino acid substitution could have on protein folding – but would not be expected to directly affect the amount of the protein, as are typically modelled with loss‐of‐function approaches. However, in this case, it was subsequently shown that the missense mutation does primarily affect the amount of ATG16L1 protein within cells, rather than the function of the protein *per se*, as the alanine substitution makes ATG16L1 protein more susceptible to caspase‐3‐mediated degradation.^30^ This in turn reduces ATG16L1 levels, and hence autophagy, during periods of cellular stress (e.g. inflammation) and results in defective clearance of intracellular pathogens and increased inflammatory cytokine production.^30^ A similar effect on intracellular pathogen clearance was also reported at the *IRGM* locus. Here, the original GWAS association (rs1000113 C>T) was shown to be in perfect linkage disequilibrium with a synonymous exonic SNP (rs10065172 C>T) that is located within a binding site (‘seed sequence’) for a microRNA, miR‐196. This microRNA was found to be highly expressed within the intestinal epithelia of patients with CD, and its expression was shown to anti‐correlate with *IRGM* mRNA levels – consistent with a physiological role in repressing IRGM.^31^ Interestingly, the disease‐associated variant (T) at rs10065172 disrupted the miR‐196 seed sequence, so that miR‐196 could not bind and resulted in carriers of the CD‐associated variant being unable to repress *IRGM* mRNA levels, which led to impaired autophagy and a failure to clear intracellular bacteria.^31^ These examples, where the functional consequences of a risk allele have been elucidated, are relatively uncommon, but highlight how the study of genetic variants can uncover specific pathway defects that contribute to CD pathogenesis.

In addition to helping resolve the role of autophagy in CD pathogenesis, the identification of genetic risk variants in known autophagy genes also led to the realisation that other susceptibility genes, which had not previously been linked to autophagy, might actually be involved in this process. For example, *NOD2 –* the first CD susceptibility gene to be discovered^2^, ^3^, ^4^ – was principally thought to be an intracellular pattern recognition receptor involved in defence against bacteria,^32^ but it was subsequently shown that NOD2 also interacts with ATG16L1 during autophagosome formation.^33^, ^34^, ^35^

The identification of autophagy genes, and specifically *ATG16L1*, also helped uncover the important role that Paneth cells are thought to play in Crohn's disease. Paneth cells are located at the base of intestinal crypts and are known to be involved in mucosal defence, producing large amounts of anti‐microbial peptides, including defensins.^36^ Early studies into the role of autophagy in intestinal inflammation revealed that mice with reduced *Atg16l1* expression had morphologically abnormal Paneth cells with reductions in the number and size of secretory granules.^37^ Similar defects were also noted in CD patients who were homozygous for the *ATG16L1* T300A variant.^38^ Interestingly, Paneth cell dysfunction has also been reported in mice following deletion of a transcription factor (*Xbp1*), which is involved in the unfolded protein response (UPR) and thereby helps to resolve ER stress.^24^ Indeed, it was subsequently shown that CD‐like ileitis spontaneously develops in mice if the UPR and autophagy were simultaneously compromised in Paneth cells, suggesting that the balance between ER stress and autophagy is likely to set an important threshold for initiating intestinal inflammation.^39^

The realisation that autophagy plays an important role in CD has also led to questions about whether pharmacological manipulation of autophagy could be used therapeutically. Studies involving mouse models have shown that rapamycin (sirolimus), which upregulates autophagy by inhibiting mTOR, can ameliorate experimental colitis.^40^, ^41^ A beneficial effect of sirolimus has also been reported in a case study of an adult with refractory CD^42^ and a retrospective case series of children with refractory IBD,^43^ although no randomised placebo‐controlled trials have yet been performed. This, however, remains an active area of research, not least because sirolimus is also known to also have anti‐fibrotic effects that could be beneficial in CD – a trial investigating this is currently underway (NCT02675153).

### The IL‐23/IL‐17 axis, bridging the innate and adaptive immune system

Early studies into the pathogenesis of IBD identified differences in the ratio of T helper (T_H_)1/T_H_2 cells in the intestinal mucosa of patients with CD or UC, and corresponding differences in their cytokine profiles.^44^, ^45^, ^46^ CD appeared to be a T_H_1‐associated disease with increased levels of interferon‐γ and IL‐12, while UC appeared to more T_H_2‐like – being characterised by increased IL‐5 and IL‐13 production (although not IL‐4). Several animal models supported this hypothesis, with an anti‐IL‐4 antibody showing therapeutic benefit in oxazolone‐induced colitis,^47^ which histologically resembles UC, while the transmural, CD‐like colitis induced by 2,4,6 trinitrobenzene sulphonic acid could be abrogated by an anti‐IL‐12 antibody^48^ – a molecule that was subsequently found to be effective in human CD trials.^49^ However, this hypothesis ultimately proved to be overly simplistic, and it was soon realised that the anti‐IL‐12 antibody would have also blocked IL‐23 due to its target antigen being a common subunit (p40) that is shared between these cytokines.^50^ Indeed, it was later shown that the effect upon IL‐23, a cytokine important in the development and maintenance of mucosal effector T_H_17 cells, was responsible for the therapeutic benefit in animal models.^51^ Activated T_H_17 cells could also be identified in the blood and intestine of CD patients.^52^ Around this time, genetics provided further support for a possible role of T_H_17 cells in CD and UC, with numerous susceptibility variants being identified within or near to genes implicated in T_H_17 biology (e.g. *RORC*,* IL23R*,* IL12B*,* TYK2*,* JAK2*,* STAT3*,* CCR6* and *ICOSLG*, Table 1).^9^, ^10^ However, while these genetic associations are consistent with a role for T_H_17 cells in CD pathogenesis, it is important to note that the functional consequences of the risk alleles at most of these loci have not yet been elucidated. As such, we still need to confirm that these genes are directly affected by the disease‐associated SNPs at these loci, and also that the functional consequences of the risk alleles specifically affect IL‐23/IL‐17 signalling, as several of these genes are also involved in other pathways (e.g. *STAT3* is critical for IL‐10 signalling). Accordingly, the precise role of T_H_17 cells in CD pathogenesis still needs to be fully characterised, and in this context, it is notable that antibodies which target a key T_H_17 cytokine, IL‐17, are ineffective in CD^53^ and yet are highly effective in other T_H_17‐driven diseases.^54^

Another reason why the exact role of T_H_17 cells remains obscure, despite several lines of evidence appearing to implicate them in CD pathogenesis, is because of the discovery of innate lymphoid cells (ILCs). These cells belong to the lymphoid lineage but do not express antigen‐specific receptors and are therefore not part of the adaptive immune system.^55^ Nonetheless, subsets of ILCs exist that mirror T‐cell subsets in terms of their cytokine secretion profiles.^55^ Type 3 ILC cells (ILC3s), for example, correspond to T_H_17 cells in that they can express RORγt, produce IL‐17 and IL‐22, and are responsive to IL‐23.^55^ Moreover, these cells have been shown to be responsible for bacteria‐driven colitis in mice that lack T cells^56^ and to selectively accumulate in the intestine of patients with active CD.^57^ Nevertheless, their specific contribution to CD pathogenesis – as distinct from that of T_H_17 cells – is difficult to ascertain because not only are they greatly outnumbered by T cells in the intestine, but many of the methods used to deplete them will also affect T cells (and vice versa) making it difficult to disentangle their relative contributions. To complicate things further, it appears that other ILC subsets may also play a role in CD pathogenesis. For example, type 1 ILCs (ILC1s), which resemble T_H_1 cells in that they express the transcription factor T‐bet, respond to IL‐12 and can produce interferon‐γ, are substantially increased in the intestine of patients with CD.^58^ For this reason, although the exact role of ILCs in IBD pathogenesis has not been fully established, this remains an active area of research – especially as they might provide tissue‐specific therapeutic targets for some patients.

### The failure to suppress aberrant immune responses

It has been estimated that the human body contains ~40 trillion bacteria, of which the vast majority are located within the intestine.^59^ This means that there are actually more bacteria in our bodies than human cells and illustrates why the intestine presents such a challenge to the immune system. In order to be able to mount an appropriate immune response to intestinal pathogens, for example, there have to also be mechanisms to prevent aberrant responses to nonpathogenic gut commensals in order to preserve health. Disrupting such control mechanisms is known to lead to colitis in animal models – for example, if the *Il10* gene is deleted^60^ or if regulatory T cells are absent.^61^ Results from GWAS studies have demonstrated that disruption of these anti‐inflammatory mechanisms is also likely to be involved in the development of CD. For example, several genes involved in IL‐10 signalling (the anti‐inflammatory cytokine which when deleted leads to colitis in animal models) lie near to GWAS hits, including *IL10*,* IL10RB*,* STAT3* and *TYK2*.^11^, ^62^ Although the functional consequences of these SNPs have not been determined, the notion that IL‐10 signalling may be defective in IBD is supported by other genetic studies. For example, private missense mutations in genes encoding components of the IL‐10 receptor (*IL10RA* or *IL10RB*) have also been shown to cause a severe, early‐onset form of IBD in consanguineous families due to abrogation of IL‐10 signalling.^63^ Other anti‐inflammatory cytokines – and genes involved in their signalling pathways – have also been identified within CD‐associated genetic loci, including *IL22* (another member of the IL‐10 cytokine family) and *SMAD3* and *SMAD7*, which are important components of TGFβ signalling. Indeed, SMAD7, which inhibits TGFβ signalling and is expressed at high levels in the intestines of patients with IBD,^64^ has been shown to be an effective therapeutic target via an antisense oligonucleotide (Mongersen) that binds to and degrades *SMAD7* mRNA.^65^

Although several cell types are known to play a role in suppressing exuberant immune responses, including M‐2 macrophages, certain ILC populations and regulatory B cells, by far the most studied immunosuppressive cell type are regulatory T cells (Tregs). Several GWAS hits also highlight the importance of Tregs in IBD pathogenesis. For example, it is known that IL‐2 signalling is critical for maintaining Treg numbers^66^ and for mediating their suppressor function via activation of STAT5,^67^ and GWASs have identified IBD‐associated loci that contain *IL2*,* IL2RA* (part of the IL‐2 receptor) and *STAT5*.^11^ Moreover, IL‐10 signalling – discussed earlier – is also important in conferring Tregs with the ability to suppress T_H_17‐mediated inflammation^68^ and in mediating that suppression.^69^ Additionally, there is a growing realisation that plasticity exists within T‐cell subsets and particularly between Tregs and T_H_17 cells, with evidence that T_H_17 cells can transdifferentiate into Tregs as inflammation resolves – a process that involves signalling through the aryl‐hydrocarbon receptor (*AHR*), which also harbours a genetic association with IBD.^12^ Further evidence of the role of Tregs in intestinal inflammation can also be found in patients who carry rare missense mutations in *FOXP3*, which encodes the master transcription factor for naturally occurring Tregs. The resulting syndrome, termed ‘immunodysregulation, polyendocrinopathy, enteropathy, X‐linked syndrome’ or ‘IPEX’,^70^ is characterised by an autoimmune enteropathy as well as several other severe autoimmune phenomena.

Based on these and other insights, Tregs are now also being studied as potential therapies in IBD. Different strategies have been proposed including inducing Tregs *ex vivo* from naïve T cells using an unrelated antigen^71^ or isolating thymically derived Tregs and expanding them before re‐infusing them back into patients.^72^ These expanded Tregs have been shown to be able to suppress the activation and proliferation of intestinal T cells from CD patients *in vitro* and to have an epigenetically stable *FOXP3* locus, theoretically limiting the possibility for T_H_17 conversion.^72^ However, full clinical trials using these approaches have yet to be performed, and it should be noted that in other disease areas, infused regulatory T cells have not shown the same efficacy as had been observed *in vitro* or in animal models.^73^

## Overlap with other immune‐mediated diseases – shared features of autoimmunity?

In addition to highlighting pathways that are likely to be important in the development of CD, GWAS has also provided insights into how the genetic architecture of CD relates to that of other autoimmune diseases.^16^ For example, while most IBD susceptibility loci are shared between CD and UC, it is notable that SNPs that are implicated in autophagy seem to be specific to CD, while SNPs that are located in, or near to, genes involved in epithelial barrier function tend to be more UC specific.^74^ Interesting overlaps have also been noted in other nonintestinal diseases. This is perhaps best exemplified by the IL‐23/IL‐17 axis, to which multiple autoimmune diseases have been linked, including psoriasis (SNPs in or near to *IL23R*,* IL12B*,* TYK2* and *STAT3*), ankylosing spondylitis (SNPs in or near to *IL23R*,* IL12B*,* TYK2* and *JAK2*) and multiple sclerosis (SNPs in or near to *IL12B*,* IL12A*,* TYK2* and *STAT3*). Overlap with some of these diseases, such as psoriasis or ankylosing spondylitis, was unsurprising since these diseases share certain clinical and pathogenic features with CD, although overlap with other diseases, such as multiple sclerosis, was less expected. However, it is important to note that interpreting this overlap is not straightforward. Indeed, just because the same gene is implicated in several diseases does not mean that common genetic haplotypes (and biological mechanisms) are responsible for these associations. For example, although both CD and psoriasis are both associated with multiple SNPs in *TYK2*, these signals are largely distinct.^11^, ^75^ Indeed, the only shared susceptibility SNP at this locus (rs12720356, a missense variant) shows discordant effects, with the risk allele for CD being protective for psoriasis. Discordant effects at the same SNP are surprisingly common in autoimmune disease genetics and present an additional challenge to understanding how genetic variation can influence disease susceptibility. For example, a SNP in *STAT3*, rs744166, is associated with both CD and multiple sclerosis, but the risk allele for CD is protective for multiple sclerosis and vice versa.^13^, ^76^ As such, while it is undoubtedly interesting that overlapping associations are detectable across distinct diseases – either at the SNP or gene level – much more work needs to be done to determine the true extent of any biological overlap. This work will be particularly important given emerging evidence that drugs targeting these ‘shared’ pathways may have very different effects between diseases, such as the anti‐IL17 antibody secukinumab, which is highly effective in psoriasis but entirely ineffective in CD.^53^, ^54^

## More work to do

Despite the successes described above, much more work needs to be done to fulfil the original goal of GWAS in Crohn's disease – to learn how genetic variation contributes to disease biology. For example, because our ability to identify disease‐associated variants has far outstripped our ability to characterise their functional effects, we still do not know the biological consequences for most of the associated variants. This bottleneck has led to the temptation to assume that we know which gene is mediating the effect at a particular locus – often because one particular gene might be involved in a familiar aspect of disease biology – but the reality is that there is often little or no evidence to support this. Indeed, in many cases, we are not even certain of which SNP – or combination of SNPs – is responsible for driving the genetic association since the SNPs included on GWAS chips are simply tags for haplotypes that may contain hundreds of associated variants (Figure 1). Fine‐mapping studies have sought to resolve the genetic associations within GWAS regions, with some success,^11^, ^17^ although strong linkage disequilibrium has prevented the identification of a single causal variant for most loci.^17^ Similarly, there have been several efforts to understand how genetic variation at a locus might affect the expression of nearby genes – especially since most GWAS hits (and their associated haplotypes) do not appear to affect the coding sequence of genes. This has commonly been done by combining genotypic data with transcriptomic data in order to identify correlations between SNP genotypes and gene expression. However, even when genetic variants are identified that correlate with gene expression (termed expression quantitative trait loci or eQTLs), strong linkage disequilibrium usually means that it is not possible to identify which of the SNPs within the haplotype is responsible for the observed effect.^77^, ^78^ As such, while these studies can be useful for identifying the genes that are affected by genotype at a particular SNP, they usually cannot identify the causal SNP within the haplotype. Moreover, these studies have revealed that many eQTL effects are only detectable in a specific cell type and/or in the presence of a specific stimulus,^77^, ^78^ meaning that a causal variant could easily be overlooked if the wrong tissue or condition is being examined.^79^

**Figure 2:**
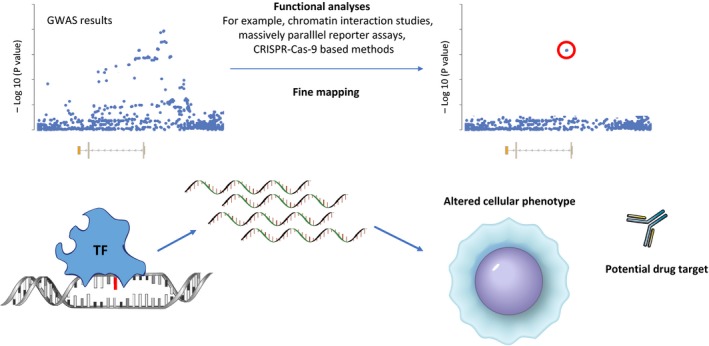
Moving from SNPs to biology. Due to linkage disequilibrium, it is not possible to know which is the causal SNP at an associated locus without additional studies such as fine‐mapping or functional analyses to systematically dissect the effects of each variant. Once this is known the mechanism by which the effect occurs and the downstream cellular consequences can be determined – for example, a SNP might introduce a binding site for a tissue‐specific transcription factor (TF) and the resulting effect on gene expression could confer an altered cellular phenotype that could provide new insights into disease biology or provide an opportunity for therapy.

For these reasons, a major goal of future studies should be to functionally dissect the consequences of disease‐associated genetic variation at each locus in order to avoid incorrectly attributing an association with a gene that is not involved and/or overlooking an important gene that is involved in an unknown aspect of disease biology because it might appear irrelevant. This is no small task, as it will need to include identification of both the cell types in which the effect is relevant and also the context (e.g. during a particular stage in development or in the presence of a particular stimulus). Examples of such studies have been performed in other fields,^80^, ^81^, ^82^ but will need to be embraced by IBD researchers in order to understand the underlying mechanisms and functional consequences of disease‐associated noncoding genetic variation (Figure 2).

Another ongoing challenge in CD genetics is to understand why the genetic variants identified to date account for only a small proportion of the variance of CD (the portion of heritability attributable to genetic variation). It has been estimated that even with such a high number of disease‐associated SNPs, we have only accounted for 26% of CD variance.^83^ The remainder, which has been termed ‘missing heritability’, still has to be accounted for, and there are several hypotheses that might explain this. First, because GWAS studies perform a univariate analysis, they cannot account for gene–gene interactions (epistasis) that might substantially alter the effect size of a given mutation. For example, in psoriasis, an epistatic interaction has been identified between genetic variants in *ERAP1* and *MHC‐C,* which has a much larger effect size that either of the variants in isolation.^84^ Second, gene–environment interactions would similarly be expected to alter the effect size attributed to a particular variant if the environmental condition specifically interacted with the pathway affected by a causal SNP. For example, tobacco smoke – a known environmental risk factor for CD – has been shown to modify the association between 64 SNPs and CD, including *NOD2* and the HLA region.^85^ Third, despite the name, most GWAS chips do not actually provide genome‐wide coverage. As such, it is likely that some of the missing heritability simply lies in regions that have not been analysed to date, either because they are difficult to impute or because they are poorly covered on GWAS chips.^15^ For instance, GWAS studies in CD have not yet analysed any SNPs on the sex chromosomes, even though these are likely to contain some disease‐associated variants.^86^ Indeed, one of the few studies to include the chromosome‐X was a meta‐analysis of 10 paediatric diseases, including CD and UC, which identified a SNP in *CD40LG*, an X chromosome gene, that was associated with CD, UC and coeliac disease.^87^ Similarly, some regions, such as the Fc receptor gene cluster or the *KIR* genes, contain highly repetitive sequences are difficult to genotype using standard approaches. Fourth, it has been proposed that rare variants – which are not included on GWAS chips but which could have much larger effect sizes – might account for some of the missing heritability. However, several studies have now been performed to attempt to identify rare disease‐associated variants, but without much success^88^, ^89^ – mirroring the situation in other diseases.^90^ Fifth, because most GWAS SNPs are proxies for the true causal variant, estimates of variance based on these are likely to underestimate the true attributable risk at each locus. With the introduction of fine‐mapping studies, though, this problem should soon be resolved.

All of these possibilities seek to address the issue of missing heritability from the perspective of identifying missing disease risk. However, it is equally possible that some of the missing heritability might be a consequence of our heritability calculations being incorrect. For instance, estimates of SNP heritability – the proportion of disease variance accounted for by risk SNPs – assume that gene–gene interactions do not occur, even though it is widely accepted that they probably do. If heritability calculations are performed using a model that allows epistasis, then it has been shown that up to 80% of the missing heritability in CD can be accounted for.^91^ Similarly, if the effects of minor allele frequency (MAF), LD and genotype certainty are factored into calculations of SNP heritability, this also tends to lead to higher estimates.^92^

## GWAS in different populations

During most of the twentieth century, the incidence of CD rose steadily in the Western world but was relatively low in developing countries.^93^ However, recently the incidence of CD in newly industrialised regions has been rising rapidly – particularly in South East Asia, South America and the Middle East – such that previously noted ethnic differences appear to be narrowing.^93^ This increase in the incidence of CD is also detectable when comparing first‐ and second‐generation immigrants from low risk areas who emigrate to countries with higher incidence of IBD^94^ and highlights the importance of environmental factors in CD development. Notably, the vast majority of GWAS studies in IBD have been performed in Caucasian populations (indeed GWAS chips were mostly designed based on Caucasian haplotype structure). Of the few studies that have been performed in individuals of non‐Caucasian descent, the results suggest that there may be some important population‐based differences in the genetic risk of CD. For example, a meta‐analysis of Asian genetic studies revealed that variants in *ATG16L1* and *NOD2*, which are associated with CD in Caucasian populations, were not associated in Han Chinese, Japanese, South Korean, Indian and Malaysian CD populations.^95^ Similarly, a GWAS in Ashkenazi Jewish CD patients identified five novel genetic regions not previously found in non‐Jewish Caucasian CD populations.^96^ A recent trans‐ethnic GWAS incorporating 86 640 individuals of European descent and 9846 individuals of East‐Asian, Indian or Iranian descent also highlighted a shared genetic risk across European and non‐European cohorts for most IBD risk loci, although genetic heterogeneity was observed – both in terms of allele frequency and/or effect size – between at several loci, including *NOD2*,* TNFSF15*,* ATG16L1*,* IL23R* and *IRGM*.^12^ In contrast, a GWAS in 2345 African Americans with IBD confirmed known risk loci but did not reveal any new variants associated with CD susceptibility.^97^ Clearly, larger studies will be necessary to better explore the commonalities and differences in the genetic variance of CD between populations, and these should ideally be performed using GWAS chips that are predicated upon the haplotype structure of the population being studied or whole genome sequencing, so as not to overlook the contribution of variants that are specific to non‐Caucasian populations.

## Pharmacogenetics

Aside from disease susceptibility, GWASs have also been used to try to identify genetic variants that are associated with specific side effects to medications commonly used to treat CD. Interest in this area was initially driven by studies in other fields that identified low‐frequency variants with large effect sizes that were responsible for well‐recognised side effects^98^ and by previous work that had demonstrated the clinical value of genotyping for mutations in *TPMT* that alter the risk of thiopurine‐induced myelosuppression.^99^ GWAS studies have since identified genetic variants in *NUDT15* that alter the risk of thiopurine‐induced myelosuppression,^100^ and in the MHC that alter the risk of thiopurine‐induced pancreatitis (HLA‐DQA1*02:01‐HLA‐DRB1*07:01)^101^ and 5‐ASA‐induced nephrotoxicity (HLA‐DRB1*03:01).^102^ Interestingly, the HLA‐DRB1*03 allele has also been associated with the development of antibodies against infliximab – an anti‐TNF‐α monoclonal antibody that is one of the most effective treatments for CD. Patients carrying the risk haplotype were almost seven times more likely to develop antibodies, which could potentially neutralise the drug and lead to a loss of efficacy.^103^

In addition to studying the genetic contribution to drug side effects, efforts have also been made to identify a genetic contribution to treatment response. So far, however, most of these studies have used a candidate gene approach, investigating individual genes or groups of genes – often those linked to CD susceptibility – rather than adopting a genome‐wide approach.^104^ Similar to disease susceptibility, it seems unlikely that a single variant will determine the efficacy of CD therapies, and therefore, a genome‐wide approach will be important. Moreover, there is no reason to suspect that variants influencing response to treatment would be the same as those that alter disease susceptibility, and thus, it was unsurprising when a polygenic risk score based on 140 CD risk loci did not associate with response to anti‐TNF therapy.^105^ To date, a genome‐wide study of response to anti‐TNF therapy has not been performed in IBD, although a study based on the Illumina immunochip (a custom designed genotyping chip containing variants that have been associated with autoimmune diseases) did provide some encouraging results,^106^ though further validation and larger independent discovery cohorts will be important. In rheumatoid arthritis (RA), such a study has already been performed and identified eight loci that were associated with response to infliximab.^107^ A similar study has also been reported in abstract form for ustekinumab, an anti‐IL12/23 antibody, in which two loci were shown to be associated with response to therapy in CD patients.^108^

In summary, although attempts to understand the genetic contribution to side effects or treatment response are far less advanced than equivalent studies in disease susceptibility, early studies have provided plenty of reason for optimism. Indeed, if genome‐wide approaches were to be adopted in suitably powered cohorts, it seems probable that genetic variants will be discovered that could both shed light on the biology responsible for responses to treatment and also potentially provide useful biomarkers for screening.

## Subphenotypes: moving beyond disease susceptibility

**Figure 3:**
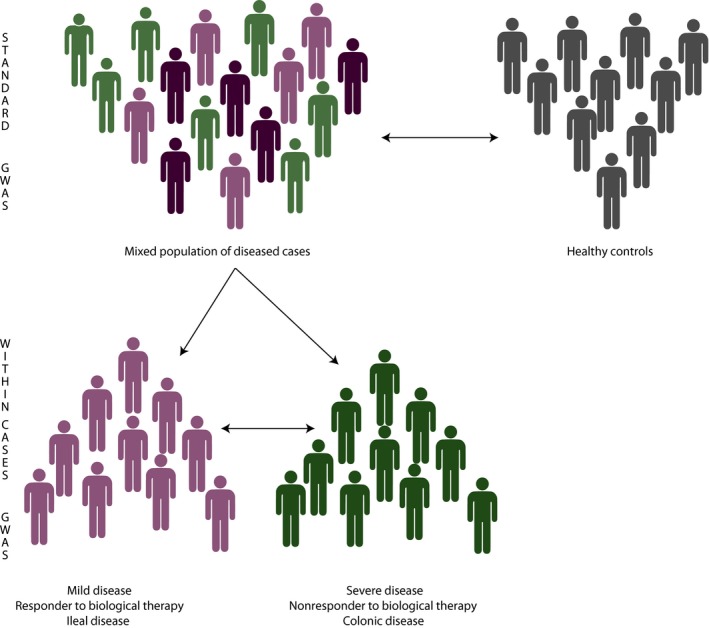
The difference between a conventional GWAS analysis and ‘within‐cases’ analysis. Schematic depicting the different analytical strategies employed by a conventional (susceptibility) GWAS in which the genetic profiles of cases and ethnically matched controls are compared, and the design of ‘within‐cases’ GWAS in which the genetic profiles of patients with distinct (and ideally contrasting) subphenotypes of a disease are compared.

Despite the success of GWAS studies into the genetic contribution to disease susceptibility, there are several other aspects of CD biology that have been proposed to have a genetic component but which – until recently – had not been studied by GWAS. For example, the distribution of Crohn's disease within the intestine and the clinical course of disease following diagnosis (prognosis) have both been shown to follow similar patterns within families^109^ but have been largely overlooked in favour of successively larger meta‐analyses into disease susceptibility. Part of the reason for this has been the assumption that susceptibility variants would also influence other aspects of disease.^110^ For example, it was assumed that the total burden of susceptibility variants would determine disease course, so that individuals who carry more risk SNPs would experience a worse prognosis. This hypothesis has been shown to be correct for qualitative traits such as height,^111^ but in CD – and other diseases – it would only be correct if susceptibility and prognosis were on a continuous spectrum and thus influenced by the same variants. If, however, this assumption is incorrect, then it would be very unlikely for SNPs, which influence disease prognosis to be identified through comparison of unstratified cases with controls.^112^ Instead, it would be necessary to directly compare subsets of patients based on phenotypic differences – a so‐called within‐cases analysis (Figure 3).

To date, there have only been a few examples of using within‐cases analyses in CD genetics. The largest study was performed by the International IBD Genetics Consortium and investigated the contribution of a large number of autoimmune disease‐associated SNPs to CD distribution, age of onset and behaviour (i.e. inflammatory, stricturing or fistulating). Although this identified little or no genetic association with disease behaviour, it did identify three loci that were associated with disease location (*NOD2*,* MHC* and *MST1*) and showed that a genetic risk score representing the sum of all known risk alleles for IBD showed a strong association with disease location.^113^

Despite the importance of CD prognosis in determining patient well‐being, most of the studies into the genetic contribution to prognosis have focused on small numbers of susceptibility SNPs in relatively small numbers of patients and have unsurprisingly provided inconsistent results.^114^, ^115^, ^116^ One of the few replicable associations identified was between variants in *NOD2* and need for surgery,^117^ although this was subsequently shown to be entirely driven by the association between *NOD2* and ileal disease (which is most commonly treated with surgery). Indeed, if this analysis is stratified by disease location, then no association between disease course and *NOD2* variants is detectable.^113^ For this reason, we elected to investigate the genetics of CD prognosis using a within‐cases analysis in which the genetic profiles of patients with contrasting courses of CD would be compared. This was first done as a candidate gene study and identified a noncoding SNP in *FOXO3* that was not a disease susceptibility variant, but which was associated with a milder course of CD^118^ – an association that has since been replicated.^119^, ^120^ Functional studies into the biological consequences of this SNP, which does not affect disease susceptibility, identified a FOXO3‐driven pathway that abrogated inflammatory responses in monocytes via TGFβ1 and led to reduced TNF‐α and IL‐6 production in carriers of the mild CD‐associated allele.^118^ Interestingly, this SNP was also shown to associate with good prognosis in RA (another TNF‐α‐driven disease^121^) and with poor prognosis in malaria (in which TNF‐α is anti‐parasitic).^118^ Since then, this variant has also been independently associated with clinical outcome in tuberculosis,^122^ another disease in which TNF‐α is known to be important. Based on this result, we then extended the analysis to a genome‐wide level and identified a further three loci that were significantly associated with prognosis in CD (*XACT*, a region upstream of *IGFBP1* and the MHC region).^120^ Strikingly, none of these variants were associated with disease susceptibility, and conversely, none of the CD susceptibility variants were associated with prognosis in our analysis, either individually or collectively. This demonstrated that the genetic contribution to prognosis comes from loci that are distinct from those that drive disease development, which in turn has important implications for our understanding of disease pathogenesis and could provide new opportunities for drug development and/or personalised medicine. In the future, larger within‐cases analyses will be important to further resolve the genetic contribution to clinically important disease subphenotypes, but the success of these will critically depend on there being detailed, high‐quality and consistent phenotypic data, else they are likely to provide (falsely) negative results.

## Conclusions

During the past decade, our understanding of CD pathogenesis has benefitted greatly from the advent of GWAS – success that has been possible thanks to large‐scale international collaboration. This has resulted in several novel insights into disease biology, which are now being investigated as therapeutic targets. However, it is important not to think that the job is done. There is much work left to do in order to fulfil the original goals of GWAS and learn more about disease biology, from determining the functional consequences at each associated locus to exploring genetic risk in other populations to identifying the genetic contribution to other important aspects of disease biology, such as prognosis. Much has been accomplished since the first CD GWAS 10 years ago, but much more should be accomplishable in the next 10 years if we make the most of the discoveries to date.
